# Homologous Recombination within Large Chromosomal Regions Facilitates Acquisition of β-Lactam and Vancomycin Resistance in Enterococcus faecium

**DOI:** 10.1128/AAC.00488-16

**Published:** 2016-09-23

**Authors:** Mónica García-Solache, Francois Lebreton, Robert E. McLaughlin, James D. Whiteaker, Michael S. Gilmore, Louis B. Rice

**Affiliations:** a Department of Medicine, Rhode Island Hospital, Warren Alpert Medical School of Brown University, Providence, Rhode Island, USA; b Departments of Ophthalmology, Microbiology and Immunology, Massachusetts Eye and Ear Infirmary, Harvard Medical School, Boston, Massachusetts, USA; c Infection Bioscience, AstraZeneca R&D Boston, Waltham, Massachusetts, USA

## Abstract

The transfer of DNA between Enterococcus faecium strains has been characterized both by the movement of well-defined genetic elements and by the large-scale transfer of genomic DNA fragments. In this work, we report on the whole-genome analysis of transconjugants resulting from mating events between the vancomycin-resistant E. faecium C68 strain and the vancomycin-susceptible D344RRF strain to discern the mechanism by which the transferred regions enter the recipient chromosome. Vancomycin-resistant transconjugants from five independent matings were analyzed by whole-genome sequencing. In all cases but one, the penicillin binding protein 5 (*pbp5*) gene and the Tn*5382* vancomycin resistance transposon were transferred together and replaced the corresponding *pbp5* region of D344RRF. In one instance, Tn*5382* inserted independently downstream of the D344RRF *pbp5* gene. Single nucleotide variant (SNV) analysis suggested that entry of donor DNA into the recipient chromosome occurred by recombination across regions of homology between donor and recipient chromosomes, rather than through insertion sequence-mediated transposition. The transfer of genomic DNA was also associated with the transfer of C68 plasmid pLRM23 and another putative plasmid. Our data are consistent with the initiation of transfer by cointegration of a transferable plasmid with the donor chromosome, with subsequent circularization of the plasmid-chromosome cointegrant in the donor prior to transfer. Entry into the recipient chromosome most commonly occurred across regions of homology between donor and recipient chromosomes.

## INTRODUCTION

Enterococcus faecium has emerged as one of the leading causes of health care-associated infections due to a combination of its high intrinsic levels of resistance to commonly used antibiotics, its remarkable genome plasticity that favors the ability to acquire *de novo* resistance when challenged with new antibiotics, and its ability to survive in diverse environments (1–4). This high prevalence of antibiotic resistance, including widespread high-level resistance to the first-line antibiotics ampicillin (Amp) and vancomycin (Van) (3, 5), presents a challenge for effective treatment of E. faecium infections.

The acquisition of resistance determinants by enterococci is mediated by a variety of mobile genetic elements, including transferable plasmids, insertion sequences (ISs), and transposons, and a high degree of recombination between strains (4, 6–8). In vancomycin-resistant E. faecalis strain V583, it has been estimated that as much as 25% of the genome has been acquired via horizontal gene transfer (9). The spread of vancomycin-resistant enterococci (VRE) is one of the major concerns in hospital settings worldwide. Vancomycin resistance results from the acquisition of transposon-associated complex operons that enable the bacteria to use modified cell wall pentapeptide precursors that bind the glycopeptide antibiotics with a lower affinity (10). There are several *van* operons that vary in the type of enzymes that they encode (11). The most widely distributed in clinical strains worldwide are *vanA* and *vanB* (12). Tn*1546*, a Tn*3*-family transposon, most commonly harbors the *vanA* operon, while Tn*5382* most commonly carries *vanB*, which is similar to conjugative transposons (13).

The Tn*5382* transposon carrying *vanB* was first reported in E. faecium C68, a multiresistant clinical isolate in which it was located immediately downstream of a penicillin binding protein 5 (*pbp5*) allele conferring a high level of resistance to ampicillin. The genetic linkage of *pbp5* and *vanB* has been identified in different strains of E. faecium isolated from different geographical regions (12, 14). This association is not universal, and the *vanB* element can insert in other regions of the chromosome or can be plasmid borne (15). C68 is a clinical isolate with broad antibiotic resistance and a large plasmid (pLRM23) implicated in increased gastrointestinal colonization (16). Transfer of the *vanB* transposon and *pbp5* was observed in association with substantial quantities of C68 genomic DNA (13, 17). Those recipient strains, however, lacked *pbp5* due to a large chromosomal deletion involving Tn*916* (18).

The precise mechanism of transfer of the *vanB* element in E. faecium has not been completely resolved. Both bona fide conjugative transposition and mobilization of large fragments of chromosomal DNA, often in association with plasmids, have been described in the literature (10, 13, 15, 19, 20). The mechanisms of transfer are better elucidated in E. faecalis than in E. faecium, in a large measure because E. faecalis is considerably more amenable to genetic manipulation, yet the clinical problem of resistance, especially resistance to ampicillin, is far greater in E. faecium than E. faecalis, making understanding of the specifics of the transfer of resistance in E. faecium a high priority. In E. faecalis, the transfer of large segments of chromosomal DNA from V583 to recipient cells has been attributed to the involvement of transferable plasmids in the donor cell that recombine with the donor chromosome across common IS elements, followed by an F-like transfer of plasmid and chromosomal DNA using the plasmid origin of transfer (21). Those investigators were able to show that virtually any segment of donor chromosome could be mobilized (21).

In the current work, we were interested in analyzing the mechanism of transfer of Tn*5382* and *pbp5* from C68 to E. faecium D344RRF, whose chromosome contains a distinct *pbp5* allele but does not contain Tn*5382* or vancomycin resistance. Our studies were designed to address four specific questions: (i) Does transfer of the *vanB* operon result in the exchange of the D344RRF *pbp5* allele for the *pbp5* allele conferring high-level ampicillin resistance from donor strain C68? (ii) Does transfer of vancomycin resistance involve transposition of Tn*5382* itself? (iii) Does integration of C68 DNA into the recipient chromosome occur by homologous recombination or by IS-mediated transposition? (iv) Is C68 plasmid pLRM23 associated with the genomic transfer?

## MATERIALS AND METHODS

### Strains and media.

E. faecium strain C68 is a vancomycin-resistant strain carrying a *vanB2* resistance element integrated into the chromosome and was originally isolated from a fecal sample from a hospitalized patient (13). E. faecium strain D344RRF is a rifampin- and fusidic acid-resistant variant of clinical isolate D344R (22). Bacteria were grown on brain heart infusion (BHI) broth or agar (Fluka, St. Louis, MO).

### Conjugation experiments.

Conjugation experiments were performed either as previously described (23) or by the cross-streak technique (24). Briefly, overnight cultures of both the donor and recipient were mixed in 15-ml conical tubes at a 1:1 ratio (200 μl each culture). After 1 h, the tubes were spun down, and 200 μl of medium was kept and plated onto nonselective BHI agar plates. Mating plates were incubated at 37°C overnight. The mixed bacteria were recovered with a loop and resuspended in 3 ml of sterile phosphate-buffered saline–2 mM EDTA and centrifuged, most of the supernatant was removed, and the cells were plated onto selective BHI agar plates with vancomycin at 25 μg/ml, fusidic acid at 25 μg/ml, and rifampin at 50 μg/ml. The plates were incubated for 3 days at 37°C. Colonies were restreaked onto identical plates to confirm resistance and isolate single colonies. Single colonies were inoculated into BHI broth with vancomycin at 25 μg/ml, fusidic acid at 25 μg/ml, and rifampin at 50 μg/ml and used to make glycerol stocks and prepare genomic DNA.

To evaluate if extracellular DNA could be implicated in the transfer, the donor and recipient cells were mixed and incubated in minimal medium salts supplemented with 0.5% glucose, 0.2 mM MgSO_4_, and 0.1 mM CaCl_2_ with or without 300 μg/ml of bovine pancreas DNase I (Roche) for 1 h at room temperature. After incubation, the mixed cells were plated onto BHI agar. To test if bacteriophages played a role in DNA transfer, we followed a previously described method (21). Briefly, 25 ml of C68 overnight growth was pelleted, and the supernatant was filter sterilized using a 0.45-μm-pore-size filter. The cell-free supernatant was diluted 2-fold with fresh BHI medium, and the conditioned medium was inoculated with D344RRF. After an overnight incubation, the cells were pelleted, resuspended in 200 μl of fresh BHI, and plated onto selection plates with vancomycin at 25 μg/ml, fusidic acid at 25 μg/ml, and rifampin at 50 μg/ml.

Broth microdilution MICs for vancomycin, ampicillin, fusidic acid, and rifampin were determined in BHI broth according to a previously published method (13).

### Serial passaging.

Transconjugant A (TC-A) and TC-B, obtained from the first mating, were subjected to serial passage with either vancomycin at 10 μg/ml, ampicillin at 12.5 μg/ml, or no selection for about 400 generations in BHI broth to evaluate if continuous selection with antibiotic had an impact on the resistance levels. The sequences of the original transconjugants and the transconjugants from the final passage for each condition were subjected to whole-genome sequencing for comparison of their sequences.

### Gene expression studies.

Frozen stocks of D344RRF, C68, and TC-A before passaging (passage 0 [P0]) and after passaging (P9 and P13) were used to inoculate an overnight BHI culture. On the next morning, the cells were diluted 1:1,000 and were grown with shaking at 37°C to an optical density at 600 nm (OD_600_) of 0.2. At that point, cultures were treated with either ampicillin, vancomycin, or no antibiotic to a final concentration of half the MIC value and were grown to an OD_600_ of 0.6 (about 4 h) with shaking. Cells were broken open with glass beads (Lysing Matrix B; MP Biomedical) using a mini-BeadBeater (BioSpec), and the RNA was purified using a Qiagen RNeasy minikit. cDNA was synthesized using a Bio-Rad iScript genomic DNA Clear cDNA synthesis kit. Quantitative PCR was carried out using a Bio-Rad iTaq universal probes kit in a multiplexed reaction in a CFX98 real-time PCR cycler. Relative gene expression was calculated using the ΔΔ*C_q_* quantification cycle (*C_q_*) method and normalized to the expression of 16S rRNA (25). To compare expression levels, we did a one-way analysis of variance using Prism (v7) software (GraphPad Software Inc.). The primers and probes used for the experiment are listed in Table S4 in the supplemental material.

### Whole-genome sequencing.

(i) Illumina MiSeq sequencing. Total DNA was extracted using a Qiagen genomic tip-100 (anion-exchange tip) (Qiagen, Valencia, CA) according to the kit manual, with minor modifications. Briefly, 6 ml of an overnight culture with vancomycin at 25 μg/ml, fusidic acid at 25 μg/ml, and rifampin at 50 μg/ml was used for each DNA sample. To break up the cells, 80 μl of 100 mg/ml lysozyme was used with 2 h of incubation at 37°C. The DNA samples were diluted to 0.3 ng/μl, and 5 μl was used for library generation using a Nextera XT DNA sample preparation kit and Nextera XT index primers (Illumina, San Diego, CA). Sufficient sample was diluted to 600 μl to provide a 15- to 20-pmol multiplexed library and sequenced on an Illumina MiSeq V2 instrument as 2×150 bp paired-end reads.

(ii) PacBio single-molecule sequencing. DNA was isolated as described above for Illumina sequencing, and 10 μg of high-quality DNA was used to make large insert libraries (10 kb) to be sequenced using the Pacific Biosciences (PacBio) RS II sequencing technology (Pacific Biosciences, Menlo Park, CA). For each sample, we used one PacBio RS II SMRT cell.

### Bioinformatics.

Illumina assemblies were performed off instrument using the CLCBio Genomics Workbench (v6.5) program (Cambridge, MA). Fastq files were trimmed for quality and minimum length (50 bp), and reads were *de novo* assembled at high stringency (length fraction = 0.9; similarity fraction = 0.99) using default mismatch/insertion/deletion costs. A summary of the genome assemblies is provided in Table S2 in the supplemental material.

PacBio genome assemblies were done using the HGAP (v3) assembly platform, with a minimum read length set to 5,000 kb and a 4% error rate allowed, by the Genomic Analysis and Bioinformatics Shared Resource, Duke University Center for Genomic and Computational Biology, Durham, NC, USA.

Gene synteny and the inferred contig order were analyzed by comparing the genomes of the parental strains (D344RRF and C68) with the fully closed genomes of E. faecium DO and E. faecium Au0004 (accession numbers ASM17439v2 and GCA_000250945.1, respectively) with the Mauve (version 2.3.1) program (26). Genome annotation was done using the RAST program (27, 28), and particular genes were manually curated.

Detection of single nucleotide variants (SNVs)/indels was accomplished by mapping reads to the parent (donor and recipient) reference assembly using the same parameters. Quality-based SNVs were detected at a minimum frequency of 95% with a minimum of 30-fold coverage using default criteria. SNVs were obtained with the Geneious (v8.1.7) program (Biomatters Ltd., New Zealand) (see Table S3 in the supplemental material). The crossover regions for donor-to-recipient DNA integration were identified by SNV and validated by PCR using Phusion high-fidelity polymerase (NEB, Ipswich, MA) and Sanger sequencing. The primers used are listed in Table S4 in the supplemental material.

### Plasmid identification.

The identification of the putative pRLM23 was done by using the PacBio assembly of C68 and TC-A and looking for a contig with the presence of the hyaluronidase (*hyl*) gene. We were able to retrieve a single 217-kb contig containing the predicted full-length pLRM23 from the C68 PacBio assembly. After the identification of the canonical pLRM23 sequence using the PacBio assemblies, we searched for the presence of genes of plasmid origin in the transconjugants by mapping the Fastq reads from D344RRF and the transconjugants versus the PacBio assembly of C68 (see Table S5C in the supplemental material) and then by comparing the regions that were unique to C68 and the transconjugants but not to D344RRF. The *hyl* gene was used as an experimental pLRM23 marker. The *hyl* gene was detected by PCR and Sanger sequencing (see Fig. S2 and Table S3 in the supplemental material).

### Accession numbers.

 The GenBank accession numbers of the 14 transconjugants are LRAR00000000 to LRBE00000000, the GenBank accession number of recipient strain D344RRF is LOQQ00000000, and the GenBank accession numbers of our laboratory stock of donor strain C68 are LRAQ00000000 and LPUE00000000.

## RESULTS

### Selection of transconjugants and whole-genome sequencing.

We performed five independent matings between C68 and D344RRF to select for transconjugants (TC) that acquired vancomycin resistance. The resistance phenotypes of the parents and the obtained transconjugants are listed in Table 1. Our transfer frequencies ranged from 10^−9^ to 10^−8^ per recipient CFU, in agreement with what was previously reported from similar experiments (13, 17). Fourteen individual transconjugants (representing about 5% of all colonies obtained after the mating) resistant to vancomycin, rifampin, and fusidic acid were randomly selected for whole-genome sequencing and further characterization. Vancomycin MICs for the different transconjugants ranged from 12.5 to 400 μg/ml, and ampicillin MICs ranged from 6.3 to 400 μg/ml (Table 2). For both antibiotics, the MICs were lower than those observed in donor strain C68, as has been reported previously (29). Interestingly, the MICs for TC-A and TC-B increased to levels comparable to those observed for the parental strains after continuous passaging for ∼400 generations, and the MIC increase was observed in passaged cells regardless of the passaging conditions (with BHI only, ampicillin, or vancomycin) (Table 2). To look for possible causes for the lower observed MICs and their further increase after passaging, cells from the final passage for each condition were collected and subjected to whole-genome sequencing, and the sequences of their genomes were compared to the sequence of the genome of the corresponding unpassaged parental transconjugant to look for SNVs or other differences. We did not identify in any of the passaged transconjugants (passaged with BHI only, ampicillin, or vancomycin) differences that would suggest that the increased vancomycin and ampicillin MICs were due to genomic changes.

**TABLE 1:** Antibiotic resistance profile of the parental strains and selected transconjugants

Strain	Resistance traits^*a*^	Origin (reference)
C68	**Amp^r^, Van^r^,** Ery^r^, Tet^r^, Str^r^, Gen^r^	Clinical isolate (13)
D344RRF	Amp^r^,^*b*^ **Rif^r^, Fus^r^,** Ery^r^, Tet^r^, Str^r^, Kan^r^	Derived from clinical isolate D344R (22)
Transconjugants	**Amp^r^, Van^r^, Rif^r^, Fus^r^,** Ery^r^, Tet^r^, Str^r^, Kan^r^	Transconjugants obtained from mating C68 and D344RRF during this work

a Amp, ampicillin; Van, vancomycin; Ery, erythromycin; Tet, tetracycline; Str, streptomycin; Gen, gentamicin; Rif, rifampin; Fus, fusidic acid; Kan, kanamycin. Boldface indicates the parental antibiotic resistance genotype that was acquired by the transconjugants.

b Intermediate resistance levels.

**TABLE 2:** MICs for parental strains and transconjugants

Strain^*a*^	MIC^*b*^ (μg/ml)			
	Van	Amp	Fus	Rif
C68	>400	>400	1.6	<0.2
D344RRF	1.6	12.5	>400	>400
TC-A P0	100	50	>400	>400
TC-A BHI P13	400	200	>400	>400
TC-A Van P13	400	>400	>400	>400
TC-A Amp P9	400	400	>400	>400
TC-B P0	200	50	>400	>400
TC-B BHI P13	400	>400	>400	>400
TC-B Van P13	400	200	>400	>400
TC-B Amp P9	400	>400	>400	>400
TC-C	25	25	200	>400
TC-D	50	25	400	>400
TC-E	50	50	200	>400
TC-F	50	100	400	>400
TC-G	25	12.5	200	400
TC-H	50	25	200	>400
TC-I	200	25	>400	>400
TC-J	200	25	>400	>400
TC-K	100	100	>400	>400
TC-L	50	6.3	>400	>400
TC-M	100	25	>400	>400
TC-N	12.5	6.3	200	200

a BHI, Van, and Amp, the strains were passaged on BHI, vancomycin, and ampicillin, respectively, for the indicated number of passages.

b Van, vancomycin; Amp, ampicillin; Fus, fusidic acid; Rif, rifampin.

**FIG 1:**
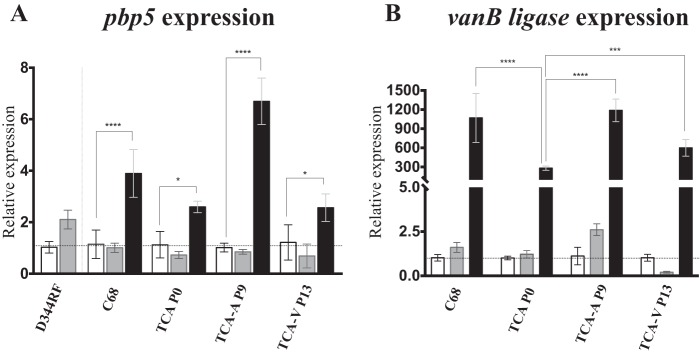
Relative expression levels of *pbp5* and *vanB* ligase in the naive TC-A transconjugant (TCA P0) or in the resulting selected ones (TCA-A P9, which was continuously passaged in the presence of ampicillin for ∼400 generations, and TCA-V P13, which was continuously passaged in the presence of vancomycin for ∼400 generations). The parental strains (D344RRF and C68) were included for comparison. The parents and the three versions of TC-A were grown with either ampicillin or vancomycin, and relative expression was calculated using the levels of expression for the corresponding group without antibiotic treatment. Expression levels of 16S rRNA were used to normalize the data. Error bars indicate the standard errors of the means for biological triplicates. (A) *pbp5* expression is not modified with respect to that for the control (untreated cells) in the presence of half the MIC of ampicillin. *pbp5* expression was induced in C68 and the three different TC-A groups in the presence of vancomycin. (B) *vanB* ligase expression was very low in the absence of vancomycin. In the presence of the antibiotic, there was a significant induction of *vanB* ligase expression. Unpassaged TC-A (P0) had lower *vanB* ligase expression levels than C68 and the passaged groups, which correlates with a lower vancomycin MIC. White bars, BHI-grown cells (calibrator); gray bars, ampicillin-grown cells; black bars, vancomycin-grown cells. ****, *P* < 0.0001; ***, *P* < 0.0002; *, *P* < 0.05.

To determine if the expression levels of *pbp5* and *vanB* ligase could be implicated in the MIC differences observed between the transconjugants and the parental strains, we used naive TC-A and its passaged derivatives, TC-A Van P13 and TC-A Amp P9, to study *pbp5* and *vanB* ligase expression in the presence of ampicillin or vancomycin. We found that in the presence of ampicillin, *pbp5* expression was not significantly modified from that in the corresponding untreated samples. In contrast, when cells were grown in the presence of vancomycin, *pbp5* expression was induced in all groups tested (Fig. 1A). The *vanB* ligase gene was minimally expressed in the absence of vancomycin and was highly induced if the cells were grown in the presence of the antibiotic. The expression levels of *vanB* ligase were significantly lower in the naive TC-A than in strain C68; after passaging, both TC-A Amp P9 and TC-A Van P13 showed increased *vanB* ligase expression that was comparable to that in C68 (Fig. 1B).

To perform in-depth analysis of the transferred DNA, we analyzed the whole-genome sequences of the 14 transconjugants (GenBank accession numbers LRAR00000000 to LRBE00000000), the recipient strain D344RRF (GenBank accession number LOQQ00000000), and our laboratory stock of donor strain C68 (GenBank accession numbers LRAQ00000000 and LPUE00000000).

We did not recover transconjugants from D344RRF cells incubated with cell-free C68 supernatant, suggesting that phage-mediated transduction is not the mechanism for DNA transfer in our system. We did recover the same proportion of transconjugants from mating reactions treated with DNase I as we did from nontreated ones, suggesting that extracellular DNA does not play a major role in DNA transfer between our two strains.

### Vancomycin resistance acquisition is associated with *pbp5* allelic replacement in E. faecium.

**FIG 2:**
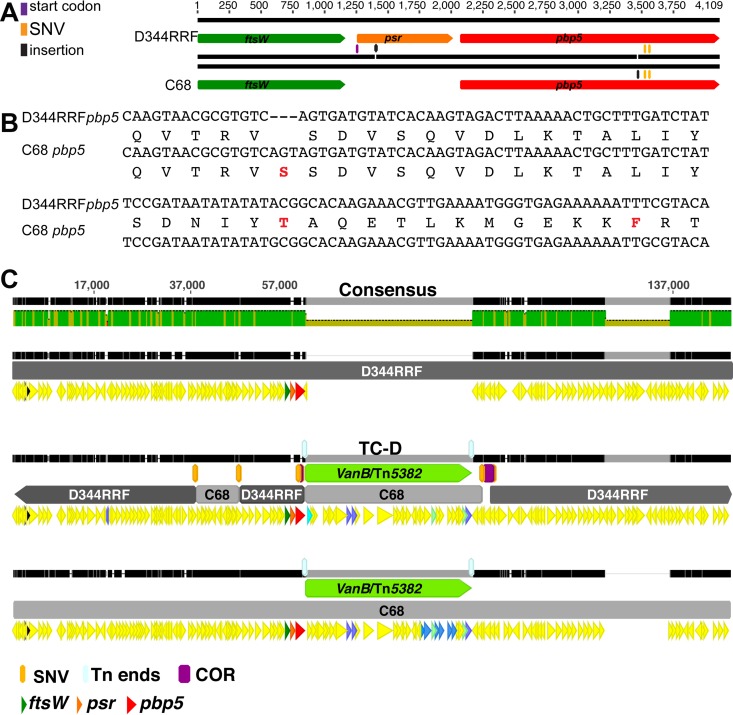
*pbp5* and TC-D insertion site. (A) *pbp5* operon cartoon showing the differences between strains D344RRF and C68. In C68, an insertion (C) 153 bp downstream of the ribosomal binding site causes a frameshift introducing a premature stop codon 309 bp downstream of the start of the gene, possibly generating a nonfunctional gene. (B) The sequence of the *pbp5* gene in C68 codes for an extra serine and has two amino acid substitutions compared with the sequence of D344RRF (red). (C) Alignment of D344RRF, TC-D, and C68 in the region of integration of Tn*5382* carrying *vanB*. Green, the vancomycin resistance-carrying transposon; light blue, the transposon (Tn) ends flanking the vancomycin resistance-carrying transposon; light gray, the segments in which the C68 genome replaced that of D344RRF in the transconjugant; purple, the crossover regions (COR) flanked by the SNV used to identify the region. Note the additional C68 integration in TC-D upstream of the *pbp5* operon. *pbp5* from D344RRF was not replaced by that of C68 in this transconjugant. SNV, single nucleotide variant.

The E. faecium *pbp5* operon consists of three genes: *ftsW*, *psr* (penicillin-binding protein synthesis repressor), and *pbp5* (29). The C68 and D344RRF *pbp5* operons differ in four positions (Fig. 2A). The first difference is the insertion of a C 153 bp downstream of the ribosomal binding site in the *psr* gene in C68, which causes a frameshift of the open reading frame and introduces a premature stop codon 309 bp downstream of the start of the gene, possibly generating a truncated protein. The second difference is the presence of an extra codon (AGT) in the C68 *pbp5* gene at position 1399, which introduces an additional serine. The other two differences are two nonsynonymous SNVs at positions 1456 and 1494, 1456A → G and 1494T → G, of the C68 *pbp5* gene (Fig. 2B). The presence of the extra serine and the two nonconservative amino acid substitutions decreases the affinity of C68 Pbp5 to penicillin (30).

Whole-genome sequencing revealed that the 14 selected transconjugants incorporated the full 34-kb region corresponding to the *vanB* resistance element Tn*5382* from the donor strain in the vicinity of *pbp5*. The ends of Tn*5382* were clearly identifiable and were conserved in all transconjugants. In none of the transconjugants did we identify the retention of *pbp5* from both C68 and D344RRF. TC-D was the only transconjugant that maintained the *pbp5* allele from recipient strain D344RRF but acquired Tn*5382* carrying *vanB*. Comparison of the sequences of TC-D and the donor and recipient strains showed that Tn*5382* integrated into the D344RRF chromosome downstream of the *pbp5* gene at a location indistinguishable from that in C68. In this region, the first SNV that distinguished D344RRF from C68 occurred 546 bp upstream of the *pbp5* stop codon. On the opposite end of the transposon, the first SNV that distinguished D344RRF from C68 occurred ca. 2 kb from the transposon end. The SNV in this location corresponded to the sequence from C68. The next SNV occurred roughly 2.4 kb from the first SNV and corresponded to the sequence from D344RRF. Interestingly, we identified an additional independent recombination event 13.8 kb further to the left of the transposon insertion that replaced approximately 9.1 kb of the recipient genome (Fig. 2C; Table 3). We identified another transconjugant (TC-M) that also had an additional recombination upstream of Tn*5382* carrying *vanB*.

**TABLE 3:** Integration site and size of integrated DNA in the studied transconjugants

TC	Position of integration site^*a*^		Size (kb) of integrated DNA			Additional integration site
	First SNV of COR^*b*^ left of *vanB*	First SNV of COR right of *vanB* (integrase site)	Minimum amt transferred^*c*^	COR between the two delimiting SNVs		
				Left	Right	
TC-A	−49.87	+100.8	184.6	574	307	
TC-B	−10.6	+49.9	94.5	3,112	4,315	
TC-C	−16.7	+27.7	78.4	6,229	1,159	
TC-D	0	+2.9	36.9	654	2,466	9,098^*d*^
TC-E	−14.9	+15.1	64	2,005	2,171	
TC-F	−10.9	+47.9	92.8	3,112	2,049	
TC-G	−61.8	+27.6	123.4	2,878	1,157	
TC-H	−60.2	+81.3	175.5	593	126	
TC-I	−16.9	+124	174.9	6,182	11	
TC-J	−16.9	+79.9	130.8	6,182	1,240	
TC-K	−10.9	+78.7	123.6	3,112	185	
TC-L	−16.9	+48.4	99.3	6,182	947	
TC-M	−10.9	+36	80.9	3,113	7,373	11,719^*e*^
TC-N	−14.9	+5.9	54.8	2,005	371	

a Considering the first position of Tn*5382* to the left side (negative values) and the last position of Tn*5382* to the right side (positive values).

b COR, crossover region.

c Including the 34-kb Tn*5382* element harboring *vanB*.

d Position −14 left of *vanB*.

e Position −16.9 of *vanB*.

### Transposition of Tn*5382* itself is not necessary for acquisition of vancomycin resistance.

We found that, unlike the case of typical conjugative transposons (31, 32), *vanB* insertion did not occur in a random or semirandom manner in the recipient's genome but occurred in association with the *pbp5* locus. In all transconjugants but one (93%), variable quantities of contiguous DNA, including the C68 *pbp5* allele, were also transferred, which is not in tune with conjugative transposition. The amount of chromosomal DNA transferred along with Tn*5382* varied in each transconjugant and ranged from 37 kb to 185 kb (Table 3). We did not find evidence of mutations in Tn*5382* that might be responsible for defective conjugative transposition.

### Integration of acquired DNA into the recipient chromosome occurs by recombination along homologous regions.

The crossover regions between the donor and recipient were identified by SNV analysis of our genome assemblies and were confirmed by PCR and Sanger sequencing.

In ca. 64% of cases (with TC-A, TC-D, TC-G, TC-H, and TC-I [downstream region] being the exceptions), the crossovers occurred in regions devoid of putative transposable elements, suggesting that the DNA integration from the donor into the recipient occurred by recombination across regions of homology and not by IS element-mediated transposition. The upstream crossover point of TC-G occurred in the region of transposon Tn*916* (18) in the D344RRF chromosome, disrupting the genes coding for the transposase and the conjugation proteins. In the case of TC-H, we were unable to confirm by PCR the crossover regions, as they are presumably within an IS element that prevented amplification.

**FIG 3:**
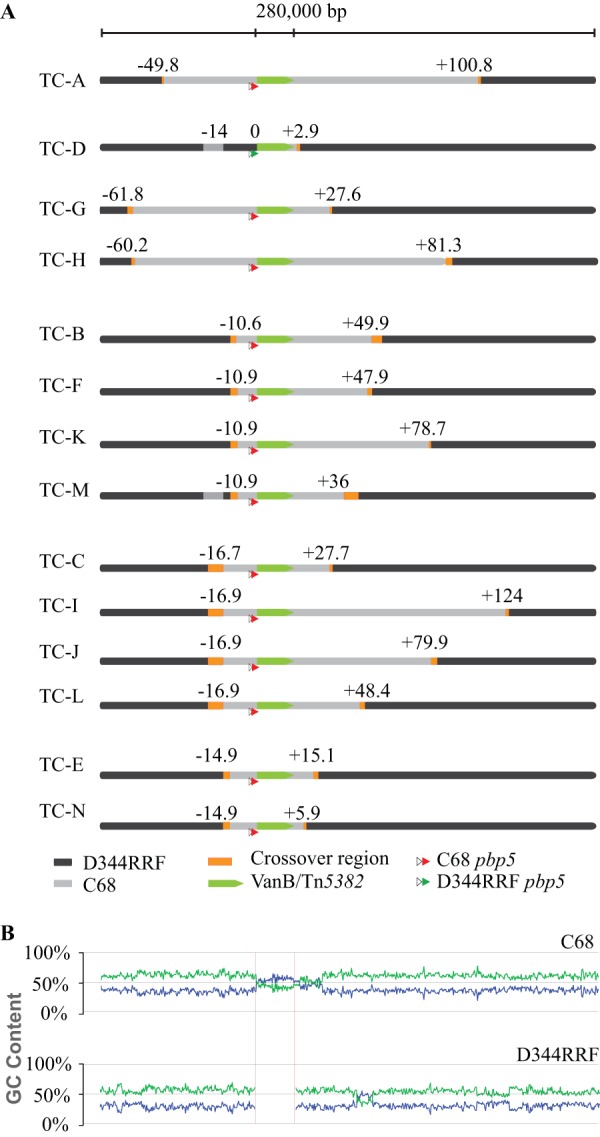
Crossover regions. (A) Cartoon representation of crossover regions in all individual transconjugants. The area of the crossover is measured from the last SNV corresponding to the donor strain, C68, to the first SNV corresponding to the recipient strain, D344RRF. Transconjugants are organized by groups with shared left crossover regions. Notice the additional crossover regions in TC-D and TC-M (light gray). The amount of chromosomal DNA integrated in addition to Tn*5382* is measured from the left or right end of the element up to the last SNV between D344RRF and each transconjugant. The amount of integrated DNA is shown in kilobases. (B) Plot of GC and AT contents of C68 (donor) and D344RRF (recipient) in the chromosomal region where crossovers occurred. Blue, GC content; green, AT content. The high-GC-content area in C68 corresponding to Tn*5382* carrying *vanB* is highlighted between red lines.

Upstream of Tn*5382* carrying *vanB*, the crossover regions in 10 transconjugants and the two secondary integrations occurred in a 17-kb region beginning 10,646 bp to the left of Tn*5382*. Within this region we identified three groups of transconjugants that shared the same or very similar crossovers: group 1 (TC-B, -F, -K, and -M), group 2 (TC-C, -I, -J, and -L), and group 3 (TC-E and -N) (Fig. 3A).

Downstream of Tn*5382* carrying *vanB* we only found two shared crossover sites in TC-C and TC-G. In other transconjugants, we identified crossovers that occurred in the same region but that were not flanked by the same SNV; these cases were TC-B, TC-F, TC-H, TC-J, and TC-K (Fig. 3A). Only TC-A did not share any crossover region with other transconjugants. None of the transconjugants shared both crossover regions. DNA integration into the recipient's chromosome was not completely at random, as several transconjugants shared crossover regions. In particular, the 17-kb region upstream of Tn*5382* carrying *vanB* appeared to be a hot spot for DNA integration. The average GC content for this region was 36.2%, which was not different for the next 17 kb upstream of it or between the donor and the recipient. Interestingly, in the regions surrounding the SNVs that mark the crossover regions, we identified sudden changes in the GC content and the presence of AT-rich strings. However, local changes of GC content are not a particular feature of the 17-kb region that could fully explain why it was preferentially targeted for recombination (Fig. 3B).

By analyzing the SNV density of recipient strain D344RRF and the transconjugants along the C68 chromosome, we identified that the region flanking Tn*5382* carrying *vanB* has fewer SNVs than the surrounding chromosome (see Fig. S1 in the supplemental material). Upstream of Tn*5382* carrying *vanB* we found only 17 SNVs in 33 kb, whereas we found 67 SNVs in the next 33 kb, and we found 31 SNVs in the 56 kb downstream of Tn*5382* carrying *vanB*, whereas we found 253 SNVs in the next 56 kb. The crossovers for 12 transconjugants (with TC-A and TC-I being the exception for the downstream crossover) occurred within this region of very few polymorphisms.

The presence of a highly homologous stretch of DNA with local AT-rich regions might explain the preference for recombination in this chromosomal region.

The SNV analysis did not suggest additional crossover regions in other parts of the D344RRF chromosome.

### pLRM23 associates with genomic DNA transfer.

C68 plasmid pLRM23 was previously identified to be an important participant in the cotransfer of antibiotic resistance (24). To get a better insight into the pLRM23 plasmid sequence and other putative transferable elements, we analyzed the C68 PacBio assembly (GenBank accession number LPUE00000000) for the presence of the hyaluronidase (*hyl*) gene, which was previously identified to be part of pLRM23. We identified *hyl* residing in a 217,169-bp contig. The contig size was comparable to that previously experimentally determined for pLRM23 on the basis of cesium chloride purification and agarose gel analysis (16). The putative pLRM23 sequence contains a high abundance of genes for mobile element proteins, transposases, and integrases/recombinases (see Table S5A in the supplemental material). It also contains putative replication initiation proteins A (*repA*) and B (*repB*) genes and a putative conjugation protein gene from the *traG-traD* family often identified in conjugative plasmids (33). Unlike other E. faecium large plasmids (34), pLRM23 does not carry genes involved in antibiotic resistance but contains a high abundance of genes from the phosphoenolpyruvate-dependent phosphotransferase system (PTS) family involved in carbohydrate metabolism and the putative regulation of virulence factors in bacteria (35). pLRM23 from C68 exhibited some similarity to other E. faecium plasmids, sharing 43.7% sequence identity with E. faecium DO plasmid 3, including the replication and conjugation proteins.

To investigate if pLRM23 was consistently cotransferred during conjugation, we first compared the canonical pLRM23 sequence (obtained from the C68 PacBio sequencing) to the PacBio sequencing data for TC-A (GenBank accession number LRHK00000000). We identified a single contig matching the TC-A sequence, suggesting that the full-length pLRM23 was transferred to TC-A. Then, by mapping the Illumina sequencing reads of all transconjugants in comparison with the canonical pLRM23 plasmid sequence, we were able to determine if the plasmid or regions of it cotransferred with the acquisition of vancomycin resistance. TC-A and TC-B harbored the same amount of putative plasmid sequences as C68, 217 kb. TC-G and TC-H had 185 and 166 kb, respectively, including in all four transconjugants a previously characterized 16.4-kb region containing the putative *hyl* (16, 36). The presence of the *hyl* gene was confirmed by PCR using the same DNA samples that were used for whole-genome sequencing of all transconjugants. The presence of pLRM23 in TC-A and TC-B was stable over ∼400 generations, as the two were subjected to serial passaging, and after that their whole genomes were sequenced. The sequences showed that the canonical pLRM23 sequence did not change during the course of the passaging.

Interestingly, the *hyl* gene was also amplified in TC-C, TC-E, and TC-F and very faintly in TC-I, whose genome sequences did not have the gene (or other pLRM23 fragments) (see Fig. S2A and Tables S1 and S5C in the supplemental material), suggesting that colonies of those transconjugants might have constituted a mixed population in which some cells still retained fragments of the plasmid. To confirm the PCR findings and to determine if the amplification of plasmid genes was stable in these transconjugants, we performed serial passages from the original glycerol stocks for 5 days and then repeated the PCR amplification of *hyl* DNA. *hyl* was detected weakly in TC-D, TC-F, and TC-I after overnight growth but not after 5 days of continuous culture (see Fig. S2B in the supplemental material), suggesting that if a small population containing pLRM23-derived sequences was present in the original stocks, this population did not persist. These data also suggest that pLRM23 or parts of it were likely cotransferred and just did not get fixed in these colonies.

In addition to the transfer of putative pLRM23, we identified a further 15-kb region that transferred from C68 to transconjugants TC-A to TC-E and TC-G to TC-I and that may have been an additional plasmid. This second plasmid, named pRIH77, has two bacteriocin-related genes and mobilization and replication genes but does not have putative conjugation genes (see Table S5B in the supplemental material), suggesting that it is a mobilizable but nonconjugative plasmid.

## DISCUSSION

Although individual transposons have been identified for both *vanA* and *vanB* elements (37–39), the transfer of these determinants between E. faecium strains has been associated with the movement of large segments of chromosomal DNA (10, 13, 15). The mechanisms for these transfers have never been precisely described, though in some cases they appear to involve the association of the donor chromosome with a conjugative plasmid prior to transfer (40, 41). In elegant experiments in Enterococcus faecalis, Manson and colleagues (21) described transfer events that involved chromosomal integration of the transferable plasmids pTEF1 and pTEF2 across similar IS elements and proposed a subsequent transfer event in which portions of plasmid and genomic DNA transfer to recipient strains using an Hfr-like mechanism (21), and a similar mechanism is proposed to happen in E. faecium (42); however, the precise mechanisms by which the transferred DNA entered into the recipient chromosome were not addressed in these studies.

Previous work by our group and others have found conflicting evidence regarding the nature of mobilization of Tn*5382*-associated vancomycin resistance (10, 13, 15, 19, 20). Our present results suggest that in most cases the acquisition of vancomycin resistance is not mediated by the direct transposition of Tn*5382*. In one of our studied transconjugants, TC-D, the insertion of Tn*5383* occurred immediately downstream of *pbp5*, and this transconjugant retained the *pbp5* gene from the recipient strain. These data could be explained by three possibilities. The first is direct transposition of Tn*5382* into the location downstream of *pbp5*, accompanied by homologous recombination of a small (less than 4-kb) region immediately downstream. The second is one-sided transposition on the left side of Tn*5382* and homologous recombination on the right. The final possibility would be entry by homologous recombination across regions flanking Tn*5382*, with the left-side recombination occurring across a small region (within 664 bp) of the genome. Our data do not allow us to distinguish between these possibilities, nor is enough known about enterococcal recombination to determine which one is the most likely.

Our data are consistent in two respects with the entry of donor chromosomal regions into the recipient chromosome occurring through homologous recombination. The first is that in none of the strains did we identify the coexistence of *pbp5* from both the donor and the recipient in the same cell. In most cases, the donor (C68) *pbp5* was the only one present in the genome sequence, suggesting that the presumptive pLRM23-chromosome cointegrant does not remain as such for long, as we were unable to identify this structure in any of our transconjugants, recombining with the recipient chromosome in regions directly involving the regions flanking *pbp5*. The second is that our SNV analysis suggested that the crossovers occurred in different regions, many of which contained no identifiable mobile elements.

The nature of our experiments did not allow a precise identification of crossover points in these transconjugants, since we relied on naturally occurring SNVs between the donor and the recipient, which were not evenly distributed throughout the genomes. Despite this, we did identify several transconjugants in which crossover regions appeared to be very similar, suggesting that characteristics of these regions facilitate homologous recombination. Crossover regions for 9 of the transconjugants were in locations devoid of IS elements or other identifiable mobile elements, suggesting that these putative hot spots were not based on the presence of transposable elements, and in none of the cases did we find the presence of IS elements in the vicinity of both crossover regions. We did identify sudden changes in GC content in regions surrounding the SNVs that mark the crossover regions clustered within the 17-kb region upstream of Tn*5382* carrying *vanB*, with the SNVs that mark the beginning and the end of the crossover region occurring in the vicinity of AT-rich stretches. These findings suggest that the regions in which recombination occur have local differences in GC content, including A/T strings with lower melting points. Another important finding was that the crossovers occurred in a region where both the D344RRF and C68 chromosomes are highly homologous, containing lower densities of SNVs and other polymorphisms than other regions of the chromosome, suggesting that a long stretch of highly homologous DNA is preferentially selected for recombination. Interestingly, it was previously described that the *pbp5* gene linked to Tn*5382* carrying *vanB* (13, 17) or as a part of a larger chromosomal region can be mobilized from one E. faecium strain to another to create hybrid strains (42).

Earlier work from our laboratory showed evidence for *pbp5* transfer from E. faecium C68 into *pbp5*-deficient strain D344SRF in a process that included an intermediate that was in a closed circular form in the transconjugant (17). This closed circular form was likely a cointegrant between the donor chromosome and C68 transferable plasmid pLRM23 that persisted for a time because of the absence of a homologous *pbp5* region in the recipient chromosome. Eventually, entry into the chromosome in these circumstances (which occurred independently in several transconjugants) likely involved crossover between IS elements on the recipient chromosome and plasmid. In the current study, we used a recipient strain that has the entire *pbp5* region in its chromosome, presenting extensive regions of homology with the transferred chromosomal region to facilitate crossover, in a model that more closely approximates transfer events as they may occur in nature.

The pLRM23 plasmid was previously associated with increased gastrointestinal colonization in a mouse infection model (16); however, the genes involved were not identified. Here we found that pLRM23 is highly enriched in PTS genes, including the four-gene cluster of the mannose-family PTS previously reported by Zhang and collaborators (43) to be important for murine gastrointestinal tract colonization after depletion of the endogenous microbiota. Genes from the PTS, including the mannose operon, were identified in association with mobile elements and possibly implicated in improved human colonization in clade A1 strains (44). The presence of the mannose PTS genes might help to explain the role of pLRM23 and related plasmids in gastrointestinal colonization. It is interesting, however, that both C68 and D344RRF also carry chromosomally encoded mannose PTS operons and D344RRF lacks the ability to successfully colonize the mouse gastrointestinal tract even at a high inoculum, suggesting that this capability is a complex trait (16).

It was previously observed that the transfer of vancomycin resistance from E. faecium C68 to a susceptible strain was associated at a high frequency (70%) with the acquisition of a *hyl*-positive plasmid (pLRM23) (24). Our current results showed only a 28% frequency of cotransfer of pLRM23 and vancomycin resistance, but this might be an underestimate due to the instability of pLRM23 in the D344RRF background or incomplete plasmid transfer. We identified genes corresponding to pLRM23 in the genome assemblies of 4 of 14 selected transconjugants. Interestingly, by PCR we identified the presence of the *hyl* gene sequence transiently in four additional transconjugants, suggesting that some parts of pLRM23 transferred to the recipient strain but did not integrate stably into the chromosome or persist as a separate replicon, likely in a mixed bacterial population. All four transconjugants in which pLRM23 persisted had large fragments of plasmid DNA in the genome assemblies, including the *repA* gene, suggesting that the plasmid replication machinery was also transferred. In at least TC-A and TC-B, it appears that plasmid transfer was complete. Complete plasmid transfer is inconsistent with transfer by an Hfr-like mechanism but in our view is compatible with the formation of a covalently closed circular transfer intermediate (plasmid-chromosome cointegrant) which excises from the donor chromosome carrying a portion of the chromosome and then transfers by conjugation (45). After conjugation, the persistence of the plasmid in the transconjugant would then depend upon whether the plasmid origin of replication and replicase genes were included in the transferred package. In the cases where partial plasmid sequences were detected, we could not rule out the possibility of an Hfr-like mechanism. Another possibility is that chromosome mobilization may be mediated by chromosomally encoded regions without the need for a conjugative plasmid. In this instance, chromosomal DNA transfer and plasmid transfer might be independent of each other; however, more work is necessary to test this possibility.

In conclusion, the transfer and replacement of large regions of genomic DNA between E. faecium strains appear to be commonly facilitated by recombination across regions of homology. A better understanding of these transfer events will inform analyses of the molecular epidemiology of resistance and virulence in this species and suggest possible mechanisms by which transfer of these determinants could be interrupted.

## Supplementary Material

Supplemental material
