# Supplementary material for: Homologous Recombination within Large Chromosomal Regions Facilitates Acquisition of β-Lactam and Vancomycin Resistance in Enterococcus faecium
Source: Antimicrob Agents Chemother. 2016 Sep 23;60(10):5777–86. doi: 10.1128/AAC.00488-16 (PMC5038250; doi:10.1128/AAC.00488-16)
Supplement: Supplemental material [file supp_60_10_5777__index.html]

Homologous Recombination within Large Chromosomal Regions Facilitates Acquisition of β-Lactam and Vancomycin Resistance in Enterococcus faecium — Supplemental material 

# Homologous Recombination within Large Chromosomal Regions Facilitates Acquisition of β-Lactam and Vancomycin Resistance in Enterococcus faecium

## Supplemental material

- Supplemental file 1 -

  Supplemental Tables S1 to S4

  PDF, 159K
- Supplemental file 2 -

  Supplemental Figures S1 and S2

  PDF, 3.6M
- Supplemental file 3 -

  Supplemental Table S5: (A) pLRM23 gene content, (B) putative pRIH77 gene content, and (C) pLRM23 coverage and pRIH77 coverage in parents and transconjugants.

  XLSX, 52K
