# Supplementary material for: Homologous Recombination within Large Chromosomal Regions Facilitates Acquisition of β-Lactam and Vancomycin Resistance in Enterococcus faecium
Source: Antimicrob Agents Chemother. 2016 Sep 23;60(10):5777–86. doi: 10.1128/AAC.00488-16 (PMC5038250; doi:10.1128/AAC.00488-16)
Supplement: Supplemental material [file AAC.00488-16_zac010165544so1.pdf]

Supplemental table 1

pLRM23 and pRIH77 transfer and persistence in the studied transconjugants

TC	pLRM23 DNA identified*	*hyl*+ in assembly	*hyl*+ by PCR	pRIH77 DNA identified	*bact+* by PCR
C68	227	✔	✔	✔	✔
TC-A	227	✔	✔	✔	✔
TC-B	227	✔	✔	✔	✔
TC-C	NI	✗	✔**	✔	✗
TC-D	NI	✗	✗	✔	✔
TC-E	NI	✗	✔**	✔	✔
TC-F	NI	✗	✔**	✗	✔**
TC-G	185	✔	✔	✔	✔
TC-H	166	✔	✔	✔	✔
TC-I	NI	✗	✔**	✔	✔
TC-J	NI	✗	✗	✗	✔**
TC-K	NI	✗	✗	✗	✔**
TC-L	NI	✗	✗	✗	✗
TC-M	NI	✗	✗	✗	✗
TC-N	NI	✗	✗	✗	✗

* DNA in KB

**Weak expression

NI: no putative plasmid sequences identified

Supplemental table 2

Whole Genome Assembly Summary

Illumina sequencing data

Genome	Total Reads[1]	Matched Reads	N50	Contigs	Average Coverage[2]
C68	2126619	1897736	47395	183	66
D344RRF	2203139	1924712	35830	207	53
TC-A	2527467	2284692	40456	247	71
TC-B	2671250	2410547	41990	263	70
TC-C	3537359	3212988	35869	214	121
TC-D	3751597	3458322	35834	212	127
TC-E	1542233	1428942	35791	217	54
TC-F	3299710	3045678	40461	212	116
TC-G	3520668	3190520	39644	250	117
TC-H	4808630	4447044	32295	272	153
TC-I	1573878	978814	38454	211	57
TC-J	1591722	1108042	35830	207	63
TC-K	1551997	1186554	35840	206	69
TC-L	1635590	1161898	35792	221	67
TC-M	2890937	2128221	35949	338	113
TC-N	3013882	2192401	42109	204	125

[1]Total Reads and corresponding N50 values represent the results from *de novo* assembly (see Materials and Methods for assembly parameters

[2]Average Genome Coverage is based on mapping coverage to the *Enterococcus faecium* strain DO chromosome (GenBank: NC_017960) for reference purposes and does not take into account mapping and coverage to plasmids associated with the *E. faecium* strain DO genome

Supplemental table 3

SVP comparison transconjugants vs. parents

Transconjugant	D344RRF	C68
C68	11954	23*
D344RRF	34*	11024
TC-A	293	10614
TC-B	55	10778
TC-C	64	10982
TC-D	37	10619
TC-E	47	9997
TC-F	70	10900
TC-G	89	10553
TC-H	44	9837
TC-I	293	10523
TC-J	132	10981
TC-K	501	10792
TC-L	61	11284
TC-M	69	11148
TC-N	43	10953

Minimum coverage: 30X
Minimum Variant Frequency: 95%
Maximum P-value: 10e-7
*Note: SVP identified between the raw reads and the assembled genome of the same strain were concentrated in regions with coverage close to the minimum, mostly at the ends of contigs or in regions comprising insertion sequences and other transposable elements.

Supplemental table 4

Primers used in this work to verify Cross-Over Regions, to detect putative pLRM23 plasmid genes and to perform RT-qPCR.

NAME	SEQUENCE	AMPLICON in bp	AMPLIFIES
19C-C68-F	TATACATTATTAGATATTTACACCCATTAC GTGAAAGAACTAAGAG	400	D344R C68 TC-D
19C-C68-R	GGAACGGAAATGATTAATGCAGC		
19C-C68-F2	TATACTTGTTATTTTTGGAATCTGCTGAT TTATTTCCCT	400	D344R C68 TC-D
19C-C68-R2	TACGTCGACAAAATCAAAGCCATTTCCT		
19D/26USleftF	AGTCCAACTGAATTCTTTACTGAT	396	D344R C68 TC-E TC-N
19D/26USleftR	GCGTTTTATTGCCAGTCTGA		
19D/26USrightF	GGGGATCATCGCAAACATCA	238	D344R C68 TC-E TC-N
19D/26USrightR	AAAAGAACCGCAGCGAGTG		
19DDSleftF	AGCGACTCCTAATATGAAGAGGG	281	D344R C68 TC-E
19DDSleftR	AGTGTCCTGAATTTATCTTAAGCGA		
19DDSRightF	ACGGGAAAGCAGAGATACGA	219	D344R C68 TC-E
19DDSrightR	GCCGCTAATCCGCCTAAGAT		
19FUSleftF	CGTTGAAGATAGTCTGCGTGT	240	D344R C68 TC-F
19FUSleftR	GCTCAACTCGTCTGCCATTT		
19FUSrightF	GATACAGGCCGAAGAGTTGC	390	D344R C68 TC-F
19FUSrightR	TGCTTGTTGTGTTTTCTGGTTG		
19FDSF	AGTTGGACTTTGCGAGGAAG	2355	D344R C68 TC-F
19FDSR	ATGGATAAGCATGGCCACCT		
20BUSleftF	CCCGTTCAGACATCAAAGCC	858	D344R C68 TC-G
20BUSleftR	CAATTATTTAGTTGGGTTAGTGTTTTCG		
20BUSrightF	AACATGAACGTGCAGGGAAG	745	D344R C68 TC-G
20BUSrightR	ACATTCGGTCTCTTACAAACGT		
20CUSF	GGTATCGCTGACAGTGGAGT	802	D344R C68 TC-H*
20CUSR	ATCTGCTTGTCCGATGATCT		
20CDSF	CGGTATTTTGACGATCCCTGAG	788	D344R C68 TC-H*
20CDSR	TGAAGAGAATATCCGCAACTTGA		
21USrightF	TCACCACTTATTTGTCCTTTATTGC	100	D344R C68 TC-C TC-I TC-J TC-L
21USrightR	ATACGGCGCATTGATCAACG		
23USleftF	GTTGCAGACATCAAAACACAAGA	684	D344R C68 TC-K
23USleftR	AGCTTGATGTGTTGTGCTCT		
23USrightF	GCTCCTTGGAATGGCAAACA	620	D344R C68 TC-K
23USrightR	TCGTGAAGGATCGTTGGTGT		
22/23DSleftF	AGTAGTAATCGTCGGTACAGGT	1755	D344R C68 TC-J TC-K
22/23DSleftR	CGAACTGCTCTTGTTGCCAT		
24DSF	GTCATTTCGGACCAGTCATTG	1680	D344R C68 TC-L
24DSR	CCTGCTACACCTTTGATGGC		
25DSleftF	AGAAACGAACACTTCATTGGAA	241	D344R C68 TC-M
25DSleftR	ATCCGCTAATTCTGCTTCCG		
25DSrightF	TGCAATCCTCACTTCAATCCTC	386	D344R C68 TC-M
25DSrightR	TCATAACGATAAGCATCTTGGAAGT		
26DSF	GCGGATCTAGGCGAATTGTC	560	D344R C68 TC-N
26DSR	ATCGTCCATGGAAACGCAAC		
25DSCORleftF	TGAAGGAGGTTCCGAGTATGA	313	D344R C68 TC-M
25DSCORleftR	GCTAATTCTGCTTCCGCTGT		

25DSCORrightF	GCGTGTAAAAGAACGAGATGG	411	D344R C68 TC-M
25DSCORrightR	CTTCCTTGCGCAGCTAAGAT		
DSCOP19C-F	TCAACTTCTGAGGATCTTTTCCATT	2846	D344R C68 TC-D
DSCOP19C-R	AAGCTCGATTTCAAACGGGATTG		
USCOP19C-F	GGGGATCTTATCAAGTAACGCGTGT	924	D344R C68 TC-D
USCOP19C-R	ATTAGCAGTATGGTTCAGCATATATGCGG		
DSCOP20B-F2	AACAGAAACAGCAGGACTAGTGCG	400	D344R C68 TC-C TC-G
DSCOP20B-R2	GAAAGTGGTTTTTATTGAAAGAAGGAAA TGATGCT		
DSCOP21-F	TATTTGCCAATACAAAACAGCATGTG	395	D344R C68 TC-I
DSCOP21-R	GTATGTTTGACGATTTCCCCGT		
USCOP21-F2	CTCGATAGATCTCGATCATTTTTTCTG	400	D344R C68 TC-C TC-I TC-J
USCOP21-R2	TCCGATAATCTCTTGATTACTTGTATCGAT AGA		
DSCOP23-F	CGAATTAAAAACTTTATTTCTCAATTAGA TCAGCATG	2700	D344R C68 TC-J TC-K
DSCOP23-R	ACACCGTCGATATGCGGAAGTT		
USCOP23-F	GCCAAACCAAAGCAGTCCTGT	1060	D344R C68 TC-K
USCOP23-R	GCTATCATTCCATCATTGATCGGT		
TC8USF	AAGACGTACAATGGACCGCT	792	D344R C68 TC-A
TC8USR	CAACTTCGCCAGGCAATACA		
TC8DSF	GCGATAACCACGGCTTCTTC	679	D344R C68 TC-A
TC8DSR	AGTTGAAACAGCAGCCGTTG		
TC20/25USleftF	TTCATGGTGCAACGATTCCA	625	D344R C68 TC-B TC-M
TC20/25USleftR	GCAGCATGACAGACGATACC		
TC20/25USright F	GCTCCTTGGAATGGCAAACA	620	D344R C68 TC-B TC-M
TC20/25USright R	TCGTGAAGGATCGTTGGTGT		
TC20DSleftF	TCTTTCTCGATTTGAAGCCGT	604	D344R C68 TC-B
TC20DSleftR	CACAGGATGCGGTATTTCTTCA		
TC20DSrightF	TGGATATTGACACAAGCGGT	244	D344R C68 TC-B
TC20DSrightR	CGATTTTAAAGTCCAATTGCCGT		
hyl-F	ACACACCGCATAGGA	1002	Unspecific amplification in
hyl-R	GCTAGCAATATCGA		D344RRF 1.5 kb band
Pbp5F	TGCTGCTGATGTCAAAGGAC	984	
Pbp5R	GGAGCATAACCTGTCGCAAAT		
vanWF	ATCGAAGCTGCCACAGAGAT	220	
vanWR	AGCGATTTTTCCCCTATCGT		
pbp5-qPCR F	TTTCTCTACACCGTCTGCATC		
pbp5-qPCR R	CAGGTTTGGAGATGGCTTTTG		
pbp5-qPCR probe	5'/5Cy5/TCGTGGGACAACAGGTGGGAAAT/ 3IAbRQSp/-3'		
vanB lig F	ATGATTACAGTTCCCGCAGAC		
vanB lig R	TCCAAGCACCCGATATACTTTC		
vanB lig probe	5'/56-FAM/TTCCTGCAC/ZEN/CCGATTTCGTTCCT/3IABkFQ/-3'		
16S forward	CAAGCGTTGTCCGGATTTATTG		
16S reverse	GCACTCAAGTCTCCCAGTTT		
16S probe	5'/HEX/GAGCGCAGGCGGTTTCTTAAGTCT/IAB kFQ/-3'		

* Did not amplified product in TC-H
