# Supplementary material for: Homologous Recombination within Large Chromosomal Regions Facilitates Acquisition of β-Lactam and Vancomycin Resistance in Enterococcus faecium
Source: Antimicrob Agents Chemother. 2016 Sep 23;60(10):5777–86. doi: 10.1128/AAC.00488-16 (PMC5038250; doi:10.1128/AAC.00488-16)
Supplement: Supplemental material [file AAC.00488-16_zac010165544so2.pdf]

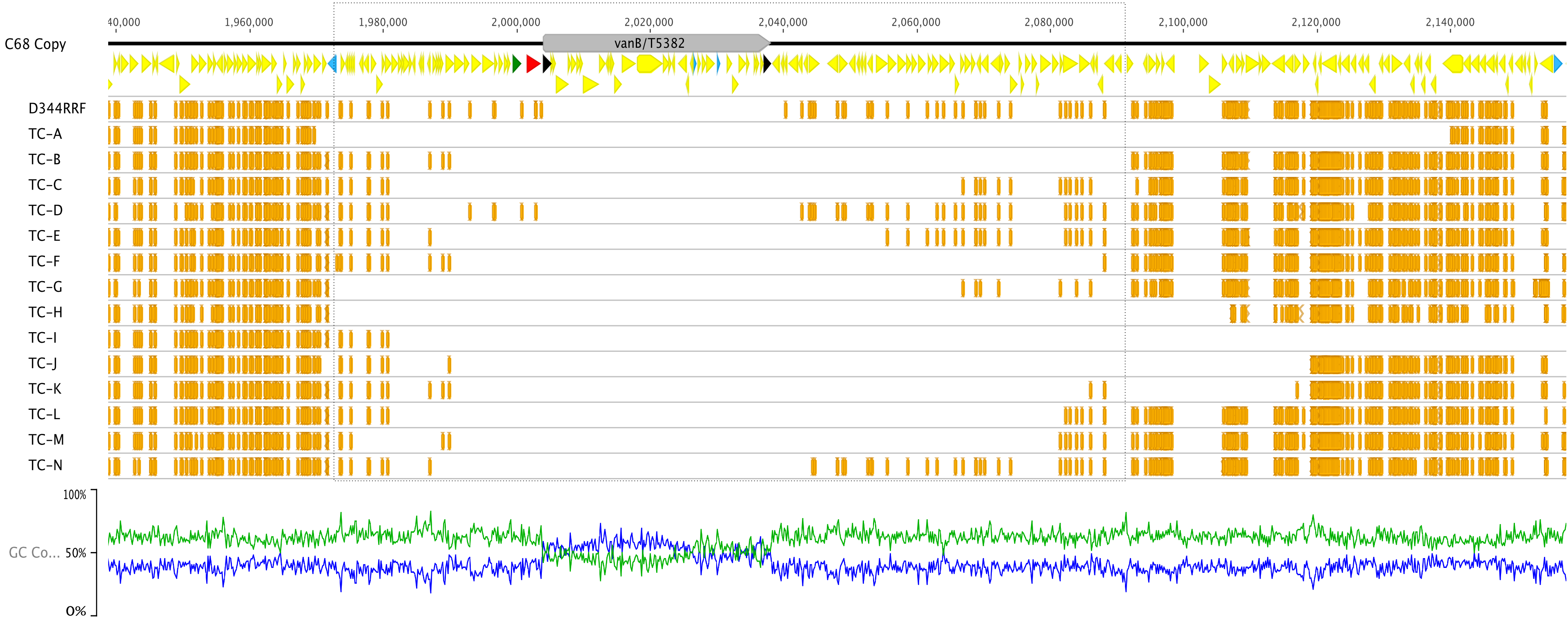

Supplemental figure 1: Schematic representation of SVP (▮) distribution along the C68 chromosome with respect to D344RRF and transconjugants TC-A to TC-N. A region of about 34 kb upstream to the left end of *vanB*/Tn*5382* and 56 kb downstream to the right end harbours less density of SVP between the C68 and the D344RRF chromosomes compared with the surroundings (dotted rectangle). Two thirds of the crossovers occurred within this region, TC-A, TC-H, TC-I, TC-J and TC-K being the exceptions. In TC-G, TC-H and TC-I one of their crossover regions is within an IS element (▶). *ftsW* ▶ ; *pbp5* ▶ ; *VanB*; ⬠ . ▶ Beginning and end of *vanB*.

The GC content is shown in blue and the AT graph is shown in green. Unlike most of the C68 genome, the *vanB* element is GC-rich (52.7%).

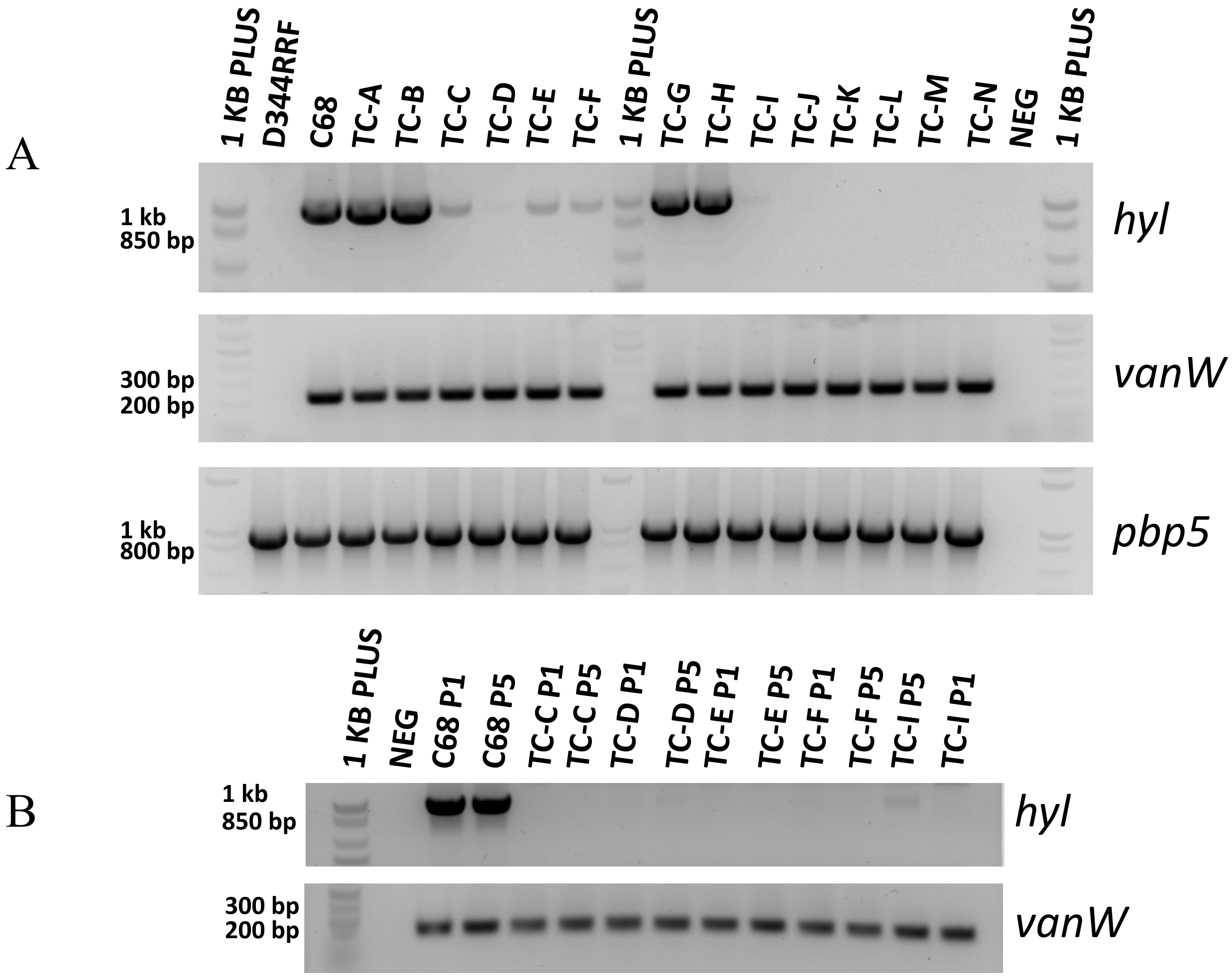

Supplemental figure 2: PCR amplification of *hyl, vanW* and *pbp5* were used as amplification controls, all samples are *pbp5* positive and all but D344RRF are *vanW* positive. **A)** DNA samples from un-passaged transconjugants as sent for sequencing. *hyl* gene is detected in C68, TC-A to TC-C, TC-E to TC-H and very faintly in TC-I. **B)** For the transconjugants that shown weak *hyl* amplification, cells were grown overnight from glycerol stocks (P1) and passaged continuously during 5 days (P5). After 5 days of continuous growth *hyl* was very weakly detected only in TC-D and TC-I.
